# Comparative Risk of Incident Coronary Heart Disease Across Chronic Inflammatory Diseases

**DOI:** 10.3389/fcvm.2021.757738

**Published:** 2021-11-10

**Authors:** Arjun Sinha, Adovich S. Rivera, Simran A. Chadha, Sameer Prasada, Anna E. Pawlowski, Edward Thorp, Matthew DeBerge, Rosalind Ramsey-Goldman, Yvonne C. Lee, Chad J. Achenbach, Donald M. Lloyd-Jones, Matthew J. Feinstein

**Affiliations:** ^1^Department of Medicine, Feinberg School of Medicine, Northwestern University, Chicago, IL, United States; ^2^Department of Preventive Medicine, Feinberg School of Medicine, Northwestern University, Chicago, IL, United States; ^3^Institute for Public Health and Management, Feinberg School of Medicine, Northwestern University, Chicago, IL, United States; ^4^Feinberg School of Medicine, Northwestern University, Chicago, IL, United States; ^5^Northwestern Medicine Enterprise Data Warehouse, Northwestern University, Chicago, IL, United States; ^6^Department of Pathology, Feinberg School of Medicine, Northwestern University, Chicago, IL, United States

**Keywords:** coronary heart disease, lupus (SLE), systemic sclerosis, inflammation, rheumatoid arthritis, HIV–human immunodeficiency virus, psoriasis

## Abstract

**Background:** Chronic inflammatory diseases (CIDs) are considered risk enhancing factors for coronary heart disease (CHD). However, sparse data exist regarding relative CHD risks across CIDs.

**Objective:** Determine relative differences in CHD risk across multiple CIDs: psoriasis, rheumatoid arthritis (RA), systemic lupus erythematosus (SLE), human immunodeficiency virus (HIV), systemic sclerosis (SSc), and inflammatory bowel disease (IBD).

**Methods:** The cohort included patients with CIDs and controls without CID in an urban medical system from 2000 to 2019. Patients with CIDs were frequency-matched with non-CID controls on demographics, hypertension, and diabetes. CHD was defined as myocardial infarction (MI), ischemic heart disease, and/or coronary revascularization based on validated administrative codes. Multivariable-adjusted Cox models were used to determine the risk of incident CHD and MI for each CID relative to non-CID controls. In secondary analyses, we compared CHD risk by disease severity within each CID.

**Results:** Of 17,049 patients included for analysis, 619 had incident CHD (202 MI) over an average of 4.4 years of follow-up. The multivariable-adjusted risk of CHD was significantly higher for SLE [hazard ratio (HR) 1.9, 95% confidence interval (CI) 1.2, 3.2] and SSc (HR 2.1, 95% CI 1.2, 3.9). Patients with SLE also had a significantly higher risk of MI (HR 3.6, 95% CI 1.9, 6.8). When CIDs were categorized by markers of disease severity (C-reactive protein for all CIDs except HIV, for which CD4 T cell count was used), greater disease severity was associated with higher CHD risk across CIDs.

**Conclusions:** Patients with SLE and SSc have a higher risk of CHD. CHD risk with HIV, RA, psoriasis, and IBD may only be elevated in those with greater disease severity. Clinicians should personalize CHD risk and treatment based on type and severity of CID.

## Introduction

Individuals living with chronic inflammatory diseases (CIDs) have an increased risk of coronary heart disease (CHD) and myocardial infarction (MI) (1, 2). In primary prevention guidelines from the European Society of Cardiology and American Heart Association, certain CIDs such as psoriasis, rheumatoid arthritis (RA), human immunodeficiency virus (HIV), and systemic lupus erythematosus (SLE) are recognized as risk-enhancing factors for atherosclerotic cardiovascular disease (ASCVD) (3, 4). Emerging data also suggest that CHD risk is elevated in other CIDs such as systemic sclerosis (SSc) and inflammatory bowel disease (IBD) (5–7). While grouping CIDs together rightly emphasizes the role of inflammation in CHD, each CID is distinct with respect to its immunologic and inflammatory dysfunction. There is also a wide range of disease severity within each CID. Thus, the degree of risk factor modification for primary prevention should match the risk of CHD based on CID type and severity. However, prior studies investigating CHD risk in inflammatory conditions either investigated only a single CID or aggregated several heterogeneous CIDs into groups (8).

Given pathophysiological and clinical heterogeneity among CIDs, which may lead to distinct patterns of CHD risk, more granular investigation of CHD risks for specific CIDs is needed. Therefore, we compared risks for incident CHD, including MI, across several disaggregated CIDs (RA, HIV, psoriasis, SLE, SSc, and IBD) in a large metropolitan health system. We also investigated associations of inflammatory phenotype severity with CHD risk within CIDs.

## Methods

### Study Population

**Figure 1:**
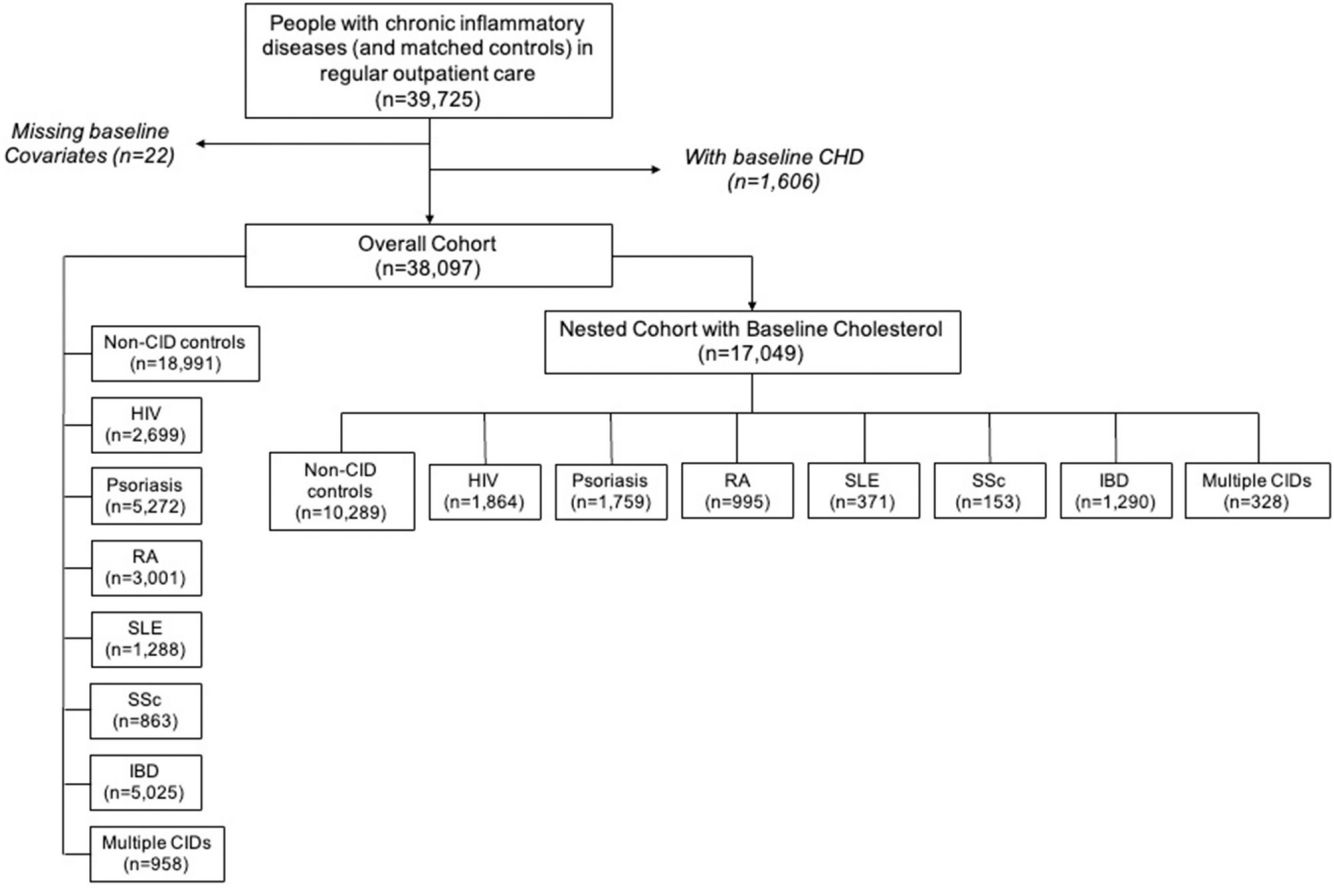
Chronic inflammatory disease cohort creation flow diagram. CHD, coronary heart disease; CID, chronic inflammatory disease; HIV, human immunodeficiency virus; RA, rheumatoid arthritis; SLE, systemic lupus erythematosus; SSc, systemic sclerosis; IBD, inflammatory bowel disease.

The Northwestern Medicine Enterprise Data Warehouse (NMEDW) was used to create an electronic health record-based cohort of persons with CIDs and non-CID controls receiving regular outpatient care in the Northwestern Medicine (NM) system, which serves the large, diverse metropolitan area of Chicago, Illinois (9). As described previously, regular outpatient care was defined as at least 1 CID specialty and/or primary care outpatient visit at least once every 2 years between baseline (the first in-person outpatient encounter) and censoring dates (death, CHD, or most recent in-person outpatient encounter at least 1 year after baseline date if more than 2 years passed between outpatient visits) (9). Controls were defined as persons in care without CIDs and were 1:1 frequency-matched with the CID population on the combination of the following variables: age, sex, race, insurance status, baseline year, hypertension, and diabetes. The overall cohort included 38,097 patients (Figure 1). However, the primary analyses were conducted in nested cohort of patients with cholesterol levels available within 1 year of baseline date, *n* = 17,049. Patients with baseline CHD and missing demographic data were excluded from the study. The cohort creation and research protocol were approved by the institutional review board at Northwestern University (Chicago, IL, USA). A waiver of informed consent was received.

### Chronic Inflammatory Disease and Controls Definitions

Adults age 18 years and older with CIDs were identified during the observation period from January 1, 2000 to January 1, 2019 using previously validated criteria: two or more International Classification of Diseases-9 (ICD-9) or ICD-10 diagnosis codes within a 2 year period were required for SSc (10), IBD (11, 12), and psoriasis (13, 14); SLE required three diagnosis codes in three separate months as described previously (15, 16); and RA required two diagnosis codes and a prescription for a disease-modifying antirheumatic drug as defined previously (17, 18). HIV was defined by validated methods including positive HIV-1 antibody or other serology, plasma HIV RNA (viral load) level at or above the lower limit of detection, and/or at least three instances in which both HIV viral load and CD4 T lymphocyte cell count/mL^3^ (CD4) were ordered concurrently (19, 20). ICD codes used as part of the above definitions are included in the Supplementary Methods. Individuals who met criteria for multiple CID diagnoses were evaluated separately.

### Key Covariates

Baseline was defined as the time of the first clinic visit with a qualifying diagnosis code. Age and data regarding sex, race/ethnicity, and insurance were obtained from the baseline visit. Baseline hypertension was defined using validated administrative codes (ICD-9 401–405 and ICD-10 I10–I15) on any date prior to 1 year after the baseline date (21, 22). Measured blood pressure values were not used to define hypertension given the heterogeneity of visit types and potential for systematic differences in measurement across exposure groups (23, 24). Baseline diabetes was defined using validated ICD-9 or ICD-10 administrative codes (ICD-9 250 and ICD-10 E10–E11, E13) and either hemoglobin A1c > 6.5% or prescription of antidiabetic medications on any date prior to 1 year after the baseline date (25). Current smoking status was obtained at baseline based on self-reported patient history. Baseline total cholesterol was obtained from the closest measurement to baseline date, within 1 year of baseline date. All data analyzed are derived from data obtained during routine clinical care, including cholesterol levels which were derived from standard clinical cholesterol assays (total cholesterol, triglycerides, high-density lipoprotein cholesterol, and calculated low-density lipoprotein cholesterol). Body-mass index (BMI) was calculated (kg/m^2^) from the closest measurements to baseline date. Deaths were confirmed by a combination of electronic health record chart review and linkage to the national death index and social security death index.

### Incident CHD

Incident CHD was defined as a new diagnosis of myocardial infarction (MI), angina, coronary revascularization, or other ischemic heart disease using validated ICD definitions, which have demonstrated high levels of agreement with expert chart review (Supplementary Methods) (26–29).

### Statistical Analyses

The period up to 90 days after baseline was treated as a blanking period due to the possibility that pre-existing CHD was not noted in the medical record until sometime shortly after baseline; therefore, people with CHD diagnosed within 90 days of the baseline date were excluded. Continuous and categorical variables were reported as mean ± standard deviation and frequency, respectively. Between-group comparisons of continuous and categorical variables were performed using ANOVA and Pearson's chi-squared tests, respectively. Follow-up period was defined as the time between baseline date and censoring date, which was the first of: (1) first encounter for CHD, (2) death, or (3) the most recent face-to-face encounter through September 9, 2019.

CHD was investigated as a composite outcome for the primary analysis. Unadjusted incidence rates for each individual CID and controls were estimated using a quasi-Poisson model to account for overdispersion of the data given varying lengths of observation periods. Cox proportional hazards models were used to analyze associations of each CID with incident CHD with non-CID controls serving as the reference group. The primary model adjusted for baseline demographics and clinical covariates including age, sex, race/ethnicity, insurance status, baseline year, diabetes, hypertension, current smoking, total cholesterol, statin use, and systemic steroid use. Secondary analyses using the same modeling approach were also performed separately with MI as the only outcome (rather than the composite of CHD, which includes MI).

We performed an exploratory analysis of incident CHD risk by disease severity within each CID. We used baseline (measured within 1 year from baseline date) C-reactive protein (CRP) levels as proxy-measures of disease severity in all CIDs except HIV, for which baseline CD4 T cell level was used as a surrogate for immune dysregulation. CD4 T cell count was used as the marker due to its known association with immune dysfunction, inflammation, and CHD in HIV (30, 31). CRP was used as the marker for the remaining CIDs as it is a clinical biomarker of general inflammation and CHD risk in the general population, as well as a marker commonly measured for a number of CIDs. We determined the risk of incident CHD for each tertile of disease severity within each CID using Cox-proportional hazard models, using the non-CID group as a standard reference group across CIDs.

After using the nested cohort with available baseline cholesterol levels for the primary analyses, we repeated our analyses in the overall cohort by removing baseline total cholesterol as an inclusion criterion to increase the available sample size. Thus, total cholesterol was not included in the model for these secondary analyses. Two-sided *p* ≤ 0.05 were considered significant. All statistical analyses were performed using R (The R Foundation for Statistical Computing) version 3.5.1.

## Results

### Clinical Characteristics

The nested cohort included 17,049 unique patients: 10,289 non-CID controls, 1,864 with HIV, 1,759 with psoriasis, 995 with RA, 153 with SSc, 371 with SLE, 1,290 with IBD, and 328 with multiple CIDs (Figure 1). One-third of the patients with multiple CIDs had either SLE or SSc. Demographics and clinical characteristics for each group in the nested cohort are presented in Table 1. There were several expected differences in demographics: patients with RA and SSc were older; patients with RA, SSc, and SLE were predominantly women while patients with HIV were predominantly men; and there were more Black adults with HIV and SLE. There were also differences in risk factors: patients with RA, SSc, and SLE had higher baseline rates of hypertension; patients with RA and psoriasis had higher baseline rates of diabetes; and the HIV cohort had the highest prevalence of active smokers. Baseline characteristics of the overall cohort (including persons without baseline cholesterol levels) are presented in Supplementary Table 1.

**Table 1:** Baseline clinical characteristics of controls and chronic inflammatory disease groups in the nested cohort.

	**Chronic inflammatory disease groups**								
	**None**	**HIV**	**Psoriasis**	**RA**	**SSc**	**SLE**	**IBD**	**Multiple CIDs**	***p*-value**
	**(*n* = 10,289)**	**(*n* = 1,864)**	**(*n* = 1,759)**	**(*n* = 995)**	**(*n* = 153)**	**(*n* = 371)**	**(*n* = 1,290)**	**(*n* = 328)**	
Age, years (mean ± SD)	50.6 ± 15.6	43.7 ± 10.7	51.5 ± 15.2	57.0 ± 13.8	55.2 ± 14.2	43.7 ± 13.7	47.9 ± 15.9	49.9 ± 13.2	<0.01
Males (%)	44.4	87.4	50.0	18.1	16.3	13.7	44.3	34.0	<0.01
Race/ethnicity (%)									<0.01
White	63.4	47.8	74.8	53.0	64.7	33.4	77.9	63.6	
Black	13.9	29.2	4.6	23.0	15.0	38.5	9.1	16.7	
Hispanic	6.6	9.3	5.7	10.2	11.8	15.1	3.7	7.4	
Asian	4.5	1.9	4.5	4.5	3.3	5.7	2.1	1.2	
Other	11.6	11.8	10.4	9.3	5.2	7.3	7.2	11.1	
Insurance (%)									<0.01
Medicaid	4.5	6.8	1.9	4.3	3.3	7.8	1.9	4.3	
Medicare	26.3	13.6	24.2	36.7	34.6	25.1	22.2	35.2	
Private	51.4	39.1	58.3	45.5	38.6	49.9	59.4	46.9	
Self-pay	17.8	40.5	15.6	13.5	23.5	17.3	16.5	13.6	
BMI, kg/m^3^ (mean ± SD)	28.0 ± 6.5	26.5 ± 5.6	29.1 ± 7.0	28.9 ± 7.4	26.2 ± 7.0	28.9 ± 7.6	27.0 ± 6.2	28.2 ± 7.2	<0.01
HTN (%)	21.6	9.4	25.8	31.8	30.7	34.2	17.0	20.4	<0.01
DM (%)	8.6	4.5	10.4	14.9	6.5	6.5	8.9	11.1	<0.01
Smoker (%)	30.1	48.3	35.1	38.7	31.4	28.8	30.1	37.0	<0.01
TC, mg/dL (mean ± SD)	188.5 ± 39.5	173.6 ± 42.1	187.2 ± 38.9	183.9 ± 39.2	174.8 ± 42.3	173.0 ± 43.2	178.0 ± 42.9	185.6 ± 38.4	<0.01
Statin use (%)	23.8	14.2	29.9	36.2	35.3	30.2	20.5	27.8	<0.01
Steroid use (%)	15.6	15.7	21.4	52.9	36.6	62.0	30.7	38.3	<0.01

*CID, chronic inflammatory disease; HIV, human immunodeficiency virus; RA, rheumatoid arthritis; SLE, systemic lupus erythematosus; SSc, systemic sclerosis; IBD, inflammatory bowel disease; SD, standard deviation; BMI, body-mass index; HTN, hypertension; DM, diabetes mellitus; TC, total cholesterol*.

### CHD Incidence Rate

There were 619 incident CHD events including 202 incident MI events over an average of 4.1 years of follow-up in the nested cohort. The crude incidence rates for CHD and MI for each CID and those with multiple CIDs in the nested cohort are described in Table 2. In the overall cohort, there were 1,196 incident CHD events including 451 incident MI events over an average of 4.1 years of follow-up. Incidence rates are provided in Supplementary Table 2; patterns across CIDs were largely similar as in the nested cohort.

**Table 2:** Crude coronary heart disease and myocardial infarction incidence rates per 1,000 person-years for controls and chronic inflammatory disease groups in the nested cohort.

**CID**	**CHD incidence rate, 1,000 person-years (95%CI)**	**MI incidence rate, 1,000 person-years (95%CI)**
Controls	8.0 (6.6, 9.5)	2.2 (1.8, 3.3)
HIV	7.7 (5.5, 11.3)	2.9 (1.8, 5.1)
Psoriasis	7.3 (4.4, 11.7)	2.2 (1.1, 5.1)
RA	10.6 (6.6, 17.2)	3.7 (1.8, 8.0)
SLE	10.2 (4.7, 21.5)	6.6 (2.6, 16.4)
SSc	16.8 (6.2, 46.4)	4.4 (0.7, 28.5)
IBD	7.3 (4.4, 12.0)	2.2 (0.7, 5.5)
Multiple	13.9 (5.8, 34.0)	4.0 (0.7, 19.7)

*CHD, coronary heart disease; MI, myocardial infarction; CID, chronic inflammatory disease; HIV, human immunodeficiency virus; RA, rheumatoid arthritis; SLE, systemic lupus erythematosus; SSc, systemic sclerosis; IBD, inflammatory bowel disease*.

### Risk of Incident CHD and MI Across CIDs

**Figure 2:**
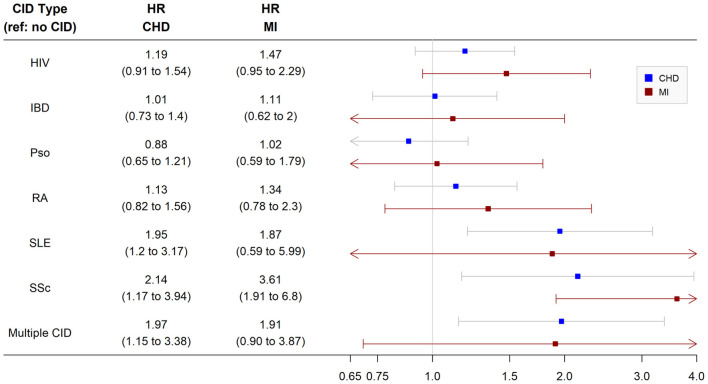
Risk of incident coronary heart disease and myocardial infarction among chronic inflammatory disease groups. Analysis performed in nested cohort. Cox proportional hazard ratios adjusted for age, sex, race/ethnicity, insurance, baseline year, hypertension, diabetes, current smoking, total cholesterol, statin use, and systemic steroid use. CHD, coronary heart disease; MI, myocardial infarction; HR, hazard ratio; CID, chronic inflammatory disease; HIV, human immunodeficiency virus; RA, rheumatoid arthritis; SLE, systemic lupus erythematosus; SSc, systemic sclerosis; IBD, inflammatory bowel disease.

In the nested cohort, we observed a significantly higher risk of incident CHD in patients with SLE (HR 2.0, 95% CI 1.2, 3.2), SSc (HR 2.1, 95% CI 1.2, 3.9), and multiple CIDs (HR 2.0, 95% CI 1.2, 3.4) compared with non-CID controls after multivariable adjustment. Patients with HIV, psoriasis, RA, or IBD did not have a significantly higher risk of incident CHD than non-CID controls (Figure 2). When the analysis was repeated in the larger overall cohort after removing baseline cholesterol from the inclusion criteria, the findings were similar (Supplementary Figure 1). Patients with SLE, SSc, and multiple CIDs had a significantly higher risk of incident CHD compared with controls after adjustment.

In secondary analyses of the nested cohort using the MI-only endpoint, only patients with SLE had a significantly higher multivariable-adjusted risk of incident MI (HR 3.6, 95% CI 1.9, 6.8) than non-CID controls. Other groups including SSc and multiple CIDs did not have a significantly higher risk of incident MI (Figure 2). When the analysis was repeated in the larger overall cohort after removing baseline cholesterol from the inclusion criteria, patients with SLE as well as SSc, HIV, and multiple CIDs had a significantly higher risk of incident MI than non-CID controls after adjustment (Supplementary Figure 1). Of note, there were no significant interactions between CID and sex or race/ethnicity with respect to CHD or MI in the nested or overall cohort.

### Inflammation and Risk of Incident CHD and MI

We next investigated associations of levels of inflammation within CID groups with risk of incident CHD and MI. Patients with HIV were divided into tertiles based on baseline CD4 T cell levels and the remaining CIDs were divided into tertiles based on baseline CRP levels. The risk of incident CHD was assessed in both the nested and overall cohorts. The risk of incident MI was assessed only in the overall cohort given insufficient MI events in the nested cohort with available CRP levels. In addition, individuals with multiple CIDs were excluded from this analysis given the use of different biomarkers for HIV (CD4 T cell count) and the other CIDs (CRP).

**Figure 3:**
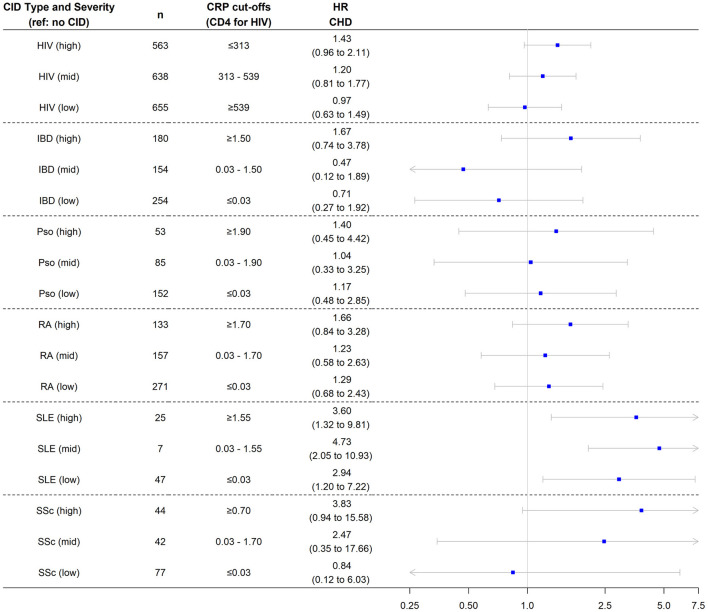
Risk of incident coronary heart disease among chronic inflammatory disease groups stratified by disease severity. Analysis performed in nested cohort. Cox proportional hazard ratios adjusted for age, sex, race/ethnicity, insurance, baseline year, hypertension, diabetes, current smoking, total cholesterol, statin use, and systemic steroid use. CHD, coronary heart disease; HR, hazard ratio; CID, chronic inflammatory disease; HIV, human immunodeficiency virus; RA, rheumatoid arthritis; SLE, systemic lupus erythematosus; SSc, systemic sclerosis; IBD, inflammatory bowel disease.

**Figure 4:**
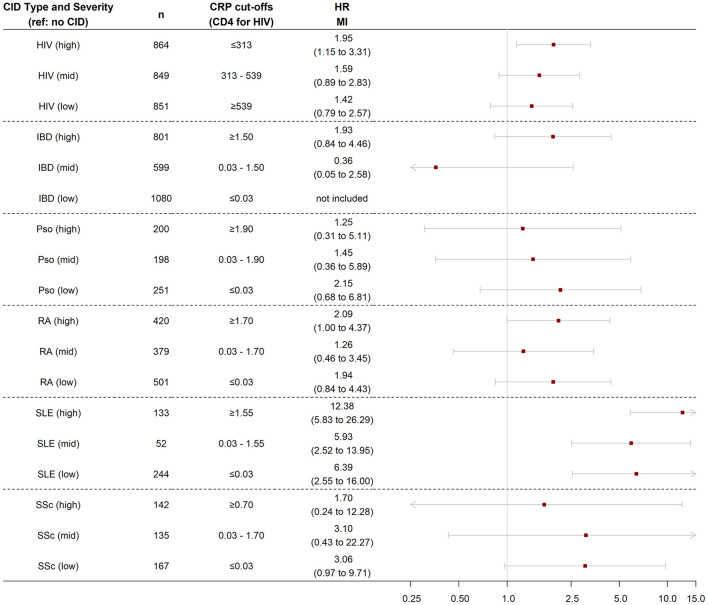
Risk of incident myocardial infarction among chronic inflammatory disease groups stratified by disease severity. Analysis performed in nested cohort. Cox proportional hazard ratios adjusted for age, sex, race/ethnicity, insurance, baseline year, hypertension, diabetes, current smoking, statin use, and systemic steroid use. MI, myocardial infarction; HR, hazard ratio; CID, chronic inflammatory disease; HIV, human immunodeficiency virus; RA, rheumatoid arthritis; SLE, systemic lupus erythematosus; SSc, systemic sclerosis; IBD, inflammatory bowel disease.

In the nested cohort, the risk of incident CHD was significantly higher in all three groups of patients with SLE—regardless of baseline CRP level—compared with controls. While the risk of incident CHD was not significantly higher in the other CID subgroups, there was a pattern by which higher inflammatory burden (higher CRP tertile or lower CD4 tertile) was associated with numerically higher CHD risk across CIDs (Figure 3). Findings were similar in the overall cohort (Supplementary Figure 2). In the overall cohort, the risk of incident MI was also significantly higher in all three groups of patients with SLE compared with controls (Figure 4). Additionally, the risk of incident MI was significantly higher in patients with HIV and RA with proxy-markers of high inflammatory burden, with ~2-fold higher risks for MI (vs. non-CID controls) for HIV patients in the lowest CD4 tertile (HR 2.0, 95% CI 1.2, 3.3) and RA patients in the highest CRP tertile (HR 2.1, 95% CI 1.0, 4.4).

## Discussion

We determined the risks of incident CHD and MI in several distinct CIDs, enabling comparison of CHD risk between disaggregated CID subtypes. We found that patients with SLE, SSc, or multiple CIDs had ~2-fold higher risks of incident CHD than patients without CIDs. Patients with SLE also had an ~4-fold higher risk of incident MI compared with non-CID controls. Generally, there was a pattern across CIDs of higher inflammation severity being associated with higher CHD risk. Patients with HIV and RA with high baseline levels of inflammation/immune dysregulation had ~2-fold higher risks for incident MI than non-CID controls.

Our results have meaningful implications for CHD risk stratification in patients with these CIDs. Currently, primary prevention guidelines appropriately note that the pooled cohort equation for ASCVD risk estimation may underestimate risk in inflammatory conditions, explicitly mentioning HIV, RA, SLE, and psoriasis (3). However, the relative differences in risk observed in our cohort emphasize the need to individualize CHD risk assessment based on type and severity of CID. The strength of association of SLE with CHD and MI was particularly noteworthy: SLE patients had a 2-fold increased risk of CHD and 4-fold increased risk of MI relative to non-CID controls—even after multivariable adjustment—and SLE patients with higher CRP levels had a 12-fold higher MI risk compared with non-CID controls. Compared with SLE, the increased risk of CHD with SSc was similar while the increased risk of MI was smaller in magnitude. Although patients with HIV, RA, IBD, and psoriasis did not have significantly higher risk for CHD compared with non-CID controls in the overall groups, the risk of MI was 2-fold higher in patients with HIV and RA with markers of advanced immune dysregulation (lower CD4 count for HIV) and inflammation (CRP for RA).

From a clinical and epidemiological standpoint, our findings are consistent with studies evaluating isolated CIDs and CHD risk. Population-based studies have demonstrated ~2 to 3-fold increased risks of MI and ASCVD in patients with SLE and SSc (5, 6, 32, 33). Multiple studies in patients with RA have demonstrated a 1.5 to 3-fold higher risk of MI (34, 35). In patients with HIV, the risk of MI correlates with CD4 T cell count. Prior analysis from the Veterans Aging Cohort Study showed a near 2-fold increased risk of MI in patients with CD4 T cell count <200 cells/mm^3^, similar to what we observed in patients with CD4 T cell count in the lowest tertile (<312 cells/mm^3^) (36). While prior studies have demonstrated an increased risk of MI and ASCVD with psoriasis, the increased risk is primarily present in those with severe psoriasis (37–39). We likely did not observe a significantly elevated risk in our patients with psoriasis because of inclusion of individuals with mild disease. We also did not observe a significant difference in CHD risk in patients with IBD after multivariable adjustment. But for both psoriasis and IBD, we observed trends toward higher CHD risk in those with higher CRP levels, although this numerically higher risk did not meet statistical significance. These findings suggest that systemic inflammation, more commonly seen with SLE, SSc, RA, and HIV, has a stronger effect on CHD and MI risk than localized inflammation, more commonly seen with IBD and psoriasis.

Biologically, comparison across CIDs also allows for potential insights into inflammatory mechanisms that may contribute to ASCVD. Our findings suggest that patients with SLE, especially those with severe disease, may have greater plaque instability. An inflammatory pathway central to SLE pathophysiology is the production of neutrophil extracellular traps (NETs). Dysregulation in NET homeostasis contributes to persistent inflammation in many CIDs, especially SLE (40–42). NETs can contribute to plaque instability through direct endothelial damage and activation of NOD-like receptor protein 3 (NLRP3) inflammasome (43). The NLRP3 inflammasome is the primary regulator of interleukin (IL)-1β, which in turn stimulates IL-6 production (44). Targeting this pathway with therapies such as Canakinumab and colchicine has shown a reduction in cardiovascular events in high risk general population (45–47). These therapies, along with further understanding of NET dysregulation, may help refine therapeutic (mechanistic) and population (CID subtype) targets to improve cardiovascular outcomes in patients with CIDs, especially SLE.

There are important limitations to our study. This was an electronic health record-based study in a single large medical system, thus making selection bias, selection of controls, and loss to follow-up potential concerns. We sought to address this by refining the inclusion criteria to require regular outpatient follow-up, as well as frequency-matching by key demographic and clinical risk factors. We further adjusted for these matching variables (given heterogeneity between CIDs) along with current smoking, total cholesterol level, statin use (although data were limited regarding statin dose and timing), and systemic steroid use. However, we were not able to include other immunosuppressive therapies used to treat these conditions, which may impact CHD risk. Despite these cohort-related limitations, including limited size, we are not aware of a large enough prospective cohort consisting of persons of similar scale and diversity of CIDs that would enable a similar comparative analysis. Other limitations include CHD and MI event ascertainment, which was based on administrative codes; although they have been demonstrated to have high levels of agreement with expert chart review as they are more likely to be discrete episodes that can be more readily captured (29). Lastly, we used CRP levels as a proxy-marker of disease severity given its widespread available and because standardized disease severity scales based on symptoms and physical examination were not widely available across CIDs in this study.

## Conclusion

Our findings meaningfully add to prior literature and allow for relative comparison between and within each CID. CHD risk was elevated in patients with SLE and SSc, regardless of level of inflammation. Patients with severe SLE had a markedly elevated risk of MI. In contrast, the risk of MI was elevated in patients with HIV and RA with high levels of inflammation/immune dysregulation. Our study provides the initial basis to include some of these distinctions in future ASCVD primary prevention guidelines and emphasizes the need to understand inflammatory mechanisms specific to each CID that contribute to CHD and MI.

## Data Availability Statement

Dataset obtained from Northwestern Medicine Enterprise Data Warehouse. De-identified dataset is available upon request to authorized individuals. Requests to access these datasets should be directed to matthewjfeinstein@northwestern.edu.

## Ethics Statement

The studies involving human participants were reviewed and approved by Institutional review board at Northwestern University. Written informed consent for participation was not required for this study in accordance with the national legislation and the institutional requirements.

## Author Contributions

AS designed the research study and wrote the manuscript. AR performed the analyses. AP generated the dataset. SC, SP, ET, MD, RR-G, YL, CA, and DL-J contributed to the discussion and edited the manuscript. MF designed the research study and edited the manuscript. All authors contributed to the article and approved the submitted version.

## Funding

This work was supported by the American Heart Association Fellow-to-Faculty Award (16FTF31200010; PI: Feinstein). National Institutes of Health R01HL156792 (PI: Feinstein) and UL1TR001422 (PI: Lloyd-Jones) and AS was supported by the National Heart, Lung, and Blood Institute of the National Institutes of Health under award number T32HL069771. YL and RR-G are funded by National Institute of Arthritis and Musculoskeletal and Skin Diseases under award number P30 AR072579.

## Conflict of Interest

The authors declare that the research was conducted in the absence of any commercial or financial relationships that could be construed as a potential conflict of interest.

## Publisher's Note

All claims expressed in this article are solely those of the authors and do not necessarily represent those of their affiliated organizations, or those of the publisher, the editors and the reviewers. Any product that may be evaluated in this article, or claim that may be made by its manufacturer, is not guaranteed or endorsed by the publisher.
