# Supplementary material for: Comparative Risk of Incident Coronary Heart Disease Across Chronic Inflammatory Diseases
Source: Front Cardiovasc Med. 2021 Nov 10;8:757738. doi: 10.3389/fcvm.2021.757738 (PMC8631433; doi:10.3389/fcvm.2021.757738)
Supplement: Supplementary file 1 [file Table_1.DOCX]

S**UPPLEMENTAL APPENDIX**

**Supplemental** **Methods**

*Coronary Heart Disease ICD Codes*

Coronary heart disease was identified by the following ICD definitions for myocardial infarction (MI), angina, coronary revascularization, or other ischemic heart disease:

| **Data Item** | **Codes** |
| --- | --- |
| MI | ICD-9: 410.x, '412'  -or-  ICD-10: 'I2101', 'I2102', 'I2109', 'I2111', 'I2119', 'I2121', 'I2129', 'I213', 'I214', 'I219', 'I220', 'I221', 'I222', 'I228', 'I229', 'I252' |
| Ischemic Heart Disease/Angina | ICD-9: '4110', '4111', '41181', '41189', '4130', '4139', '41400', '41401', '41402', '41403', '41404', '41405', '41406', '41407', '4142', '4143', '4144', '4148', '4149'  -or-  ICD-10: 'I200', 'I208', 'I209', 'I240', 'I241', 'I248', 'I249', 'I2510', 'I25110', 'I25111', 'I25118', 'I25119', 'I255', 'I256', 'I25700', 'I25701', 'I25708', 'I25709', 'I25710', 'I25711', 'I25718', 'I25719', 'I25720', 'I25721', 'I25728', 'I25729', 'I25730', 'I25731', 'I25738', 'I25739', 'I25750', 'I25751', 'I25758', 'I25759', 'I25760', 'I25761', 'I25768', 'I25769', 'I25790', 'I25791', 'I25798', 'I25799', 'I25810', 'I25811', 'I25812', 'I2582', 'I2583', 'I2584', 'I2589', 'I259' |
| Coronary Artery Bypass Grafting | ICD-9 Procedure Codes: '3610', '3611', '3612', '3613', '3614', '3615', '3616', '3617', '3619'  -or-  ICD-10 Procedure Codes:  '0210', '0211', '0212', '0213'  -or-  CPT Procedure Codes:  '33510', '33511', '33512', '33513', '33514', '33516', '33517', '33518', '33519', '33521', '33522', '33523', '33533', '33534', '33535', '33536'  -or-  ICD-9 Dx Code: 'V4581'  -or-  ICD-10 Dx Code: 'Z951' |
| Percutaneous Coronary Intervention | ICD-9 Procedure Codes: '0066', '1755', '3601', '3602', '3605', '3606', '3607', '3609'  -or-  ICD-10 Procedure Codes:  '0270', '0271', '0272', '0273', '02C0', '02C1', '02C2', '02C3', '02H0', '02H1', '02H2', '02H3', '02C34ZZ', 'X2C0361', 'X2C1361'  -or-  CPT Procedure Codes:  '92920', '92921', '92924', '92925', '92928', '92929', '92933', '92934', '92937', '92938', '92941', '92943', '92944', '92980', '92981', '92982', '92984', '92995', '92996', 'C9600', 'C9601', 'C9602', 'C9603', 'C9604', 'C9605', 'C9606', 'C9607', 'C9608', 'G0290', 'G0291'  -or-  ICD-9 Dx Code: 'V4582'  -or-  ICD-10 Dx Code: 'Z955', 'Z9861' |

**Supplemental Results**

**Supplemental Table 1.** Baseline clinical characteristics of controls and chronic inflammatory disease groups in the overall cohort

|  | Chronic Inflammatory Disease Groups | | | | | | | |
| --- | --- | --- | --- | --- | --- | --- | --- | --- |
|  | None (n=18555) | HIV (n=2591) | Psoriasis (n=5144) | RA  (n=2917) | SSc  (n=850) | SLE (n=1218) | IBD (n=4925) | Multiple CIDs  (n=463) |
| Age, years  (mean ± SD) | 48.4 ± 16.5 | 42.7 ± 11.2 | 49.6 ± 15.8 | 56.0 ± 14.6 | 53.5 ± 13.6 | 43.1 ± 14.6 | 44.6 ± 16.3 | 48.4 ± 14.2 |
| Males (%) | 41.7 | 84.7 | 47.4 | 18.0 | 15.3 | 10.3 | 42.4 | 25.3 |
| Race/ethnicity (%) |  |  |  |  |  |  |  |  |
| White | 65.7 | 44.0 | 75.8 | 60.1 | 63.9 | 43.7 | 79.7 | 65.2 |
| Black | 11.4 | 33.0 | 3.1 | 14.3 | 10.2 | 27.5 | 5.8 | 13.2 |
| Hispanic | 7.1 | 9.0 | 5.9 | 11.3 | 9.6 | 12.7 | 4.1 | 8.9 |
| Asian | 4.4 | 1.8 | 3.6 | 3.6 | 2.6 | 5.7 | 1.9 | 1.3 |
| Other | 11.4 | 12.2 | 11.6 | 10.7 | 13.6 | 10.4 | 8.5 | 11.4 |
| Insurance (%) |  |  |  |  |  |  |  |  |
| Medicaid | 5.3 | 8.2 | 3.9 | 4.4 | 4.9 | 9.5 | 3.4 | 8.0 |
| Medicare | 23.3 | 13.1 | 21.5 | 37.7 | 29.9 | 24.8 | 18.6 | 32.0 |
| Private | 51.5 | 36.5 | 59.2 | 44.8 | 42.9 | 50.0 | 59.7 | 48.4 |
| Self-pay | 19.9 | 42.2 | 15.4 | 13.1 | 22.2 | 15.7 | 18.3 | 11.7 |
| BMI, kg/m^2^  (mean ± SD) | 27.8 ± 6.5 | 26.5 ± 5.7 | 29.3 ± 7.0 | 28.6 ± 7.2 | 26.1 ± 6.4 | 28.1 ± 7.9 | 26.3 ± 6.0 | 28.0 ± 7.4 |
| HTN (%) | 17.3 | 8.4 | 19.2 | 21.1 | 13.9 | 24.7 | 10.1 | 17.9 |
| DM (%) | 6.2 | 3.8 | 6.6 | 8.3 | 2.6 | 5.7 | 3.5 | 7.1 |
| Smoker (%) | 32.2 | 50.5 | 36.4 | 38.7 | 35.6 | 28.7 | 31.2 | 38.2 |
| Statin Use (%) | 17.9 | 11.7 | 21.5 | 24.3 | 22.1 | 17.2 | 11.8 | 18.4 |
| Steroid Use (%) | 16.0 | 15.7 | 21.5 | 52.8 | 31.2 | 59.4 | 35.0 | 42.3 |

CID: chronic inflammatory disease, HIV: human immunodeficiency virus, RA: rheumatoid arthritis, SLE: systemic lupus erythematosus, SSc: systemic sclerosis, IBD: inflammatory bowel disease.SD: standard deviation, BMI: body-mass index, HTN: hypertension, DM: diabetes mellitus

**Supplemental Table 2.** Crude coronary heart disease and myocardial infarction incidence rates per 1000 person-years for controls and chronic inflammatory disease groups in the overall cohort.

| **CID** | **CHD Incidence**  **Rate, 1000 person-years (95%CI)** | **MI Incidence**  **Rate, 1000 person-years (95%CI)** |
| --- | --- | --- |
| **Controls** | 7.7 (6.6, 8.8) | 2.2 (1.8, 3.3) |
| **HIV** | 8.8 (6.6, 11.7) | 3.6 (2.6, 5.5) |
| **Psoriasis** | 6.6 (5.1, 8.8) | 2.2 (1.5, 3.6) |
| **RA** | 9.1 (6.9, 12.0) | 3.3 (2.2, 5.1) |
| **SLE** | 11.3 (7.7, 16.4) | 6.9 (4.4, 11.0) |
| **SSc** | 11.7 (6.9, 19.7) | 5.5 (2.6, 11.0) |
| **IBD** | 4.7 (3.6, 6.9) | 1.5 (0.7, 2.6) |
| **Multiple** | 14.2 (8.4, 24.1) | 4.4 (1.8, 10.6) |

CHD: coronary heart disease, MI: myocardial infarction, CID: chronic inflammatory disease, HIV: human immunodeficiency virus, RA: rheumatoid arthritis, SLE: systemic lupus erythematosus, SSc: systemic sclerosis, IBD: inflammatory bowel disease.

**
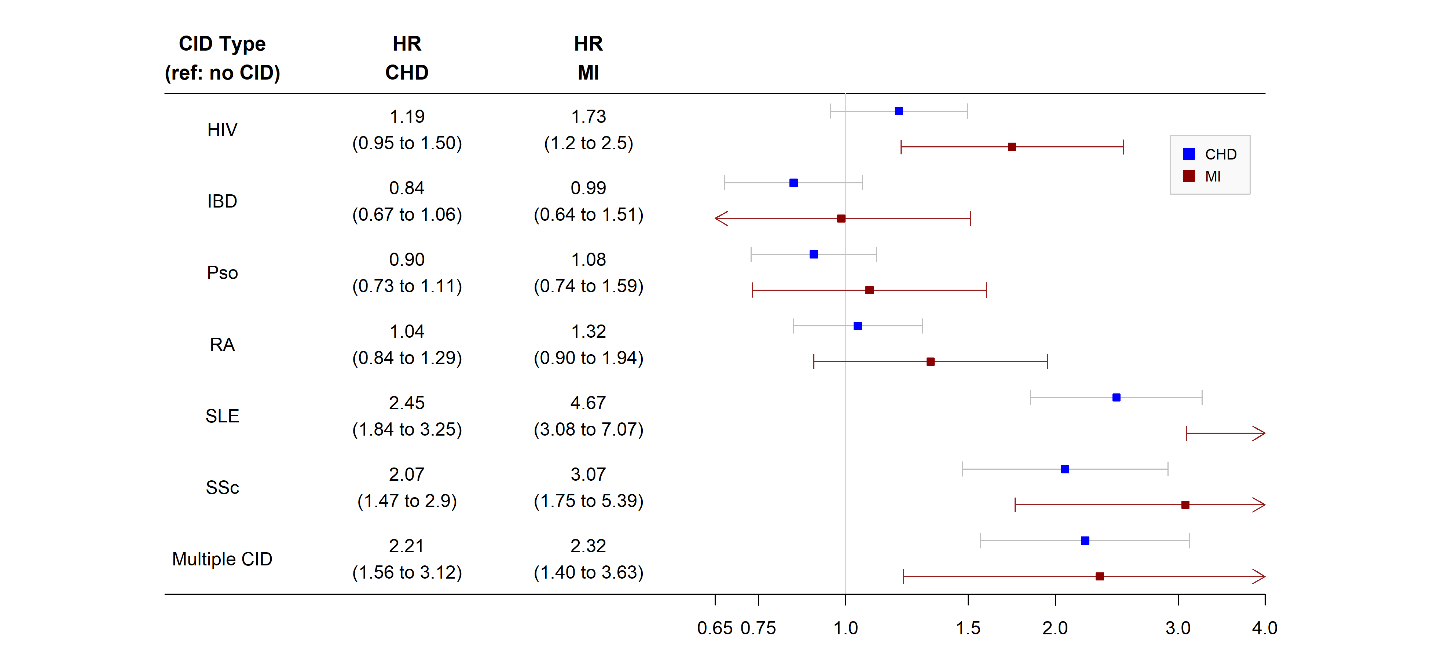
Supplemental Figure 1.** Risk of Incident Coronary Heart Disease and Myocardial Infarction among Chronic Inflammatory Disease Groups

Analysis performed in overall cohort. Cox proportional hazard ratios adjusted for age, sex, race/ethnicity, insurance, baseline year, hypertension, diabetes, current smoking, statin use, and systemic steroid use. CHD: coronary heart disease, MI: myocardial infarction, HR: hazard ratio, CID: chronic inflammatory disease, HIV: human immunodeficiency virus, RA: rheumatoid arthritis, SLE: systemic lupus erythematosus, SSc: systemic sclerosis, IBD: inflammatory bowel disease.


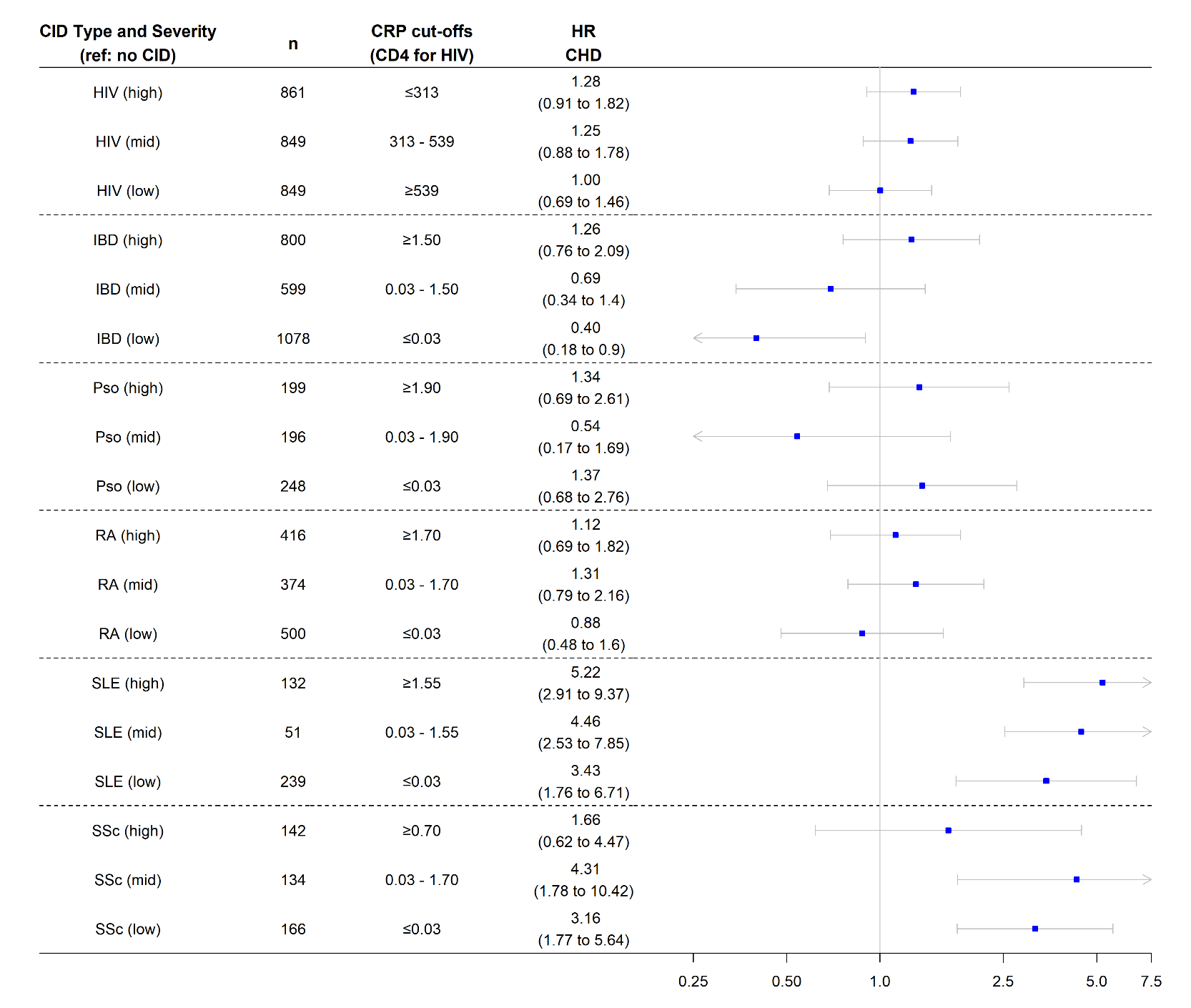
**Supplemental Figure 2.** Risk of Incident Coronary Heart Disease among Chronic Inflammatory Disease Groups Stratified by Disease Severity.

Analysis performed in overall cohort. Cox proportional hazard ratios adjusted for age, sex, race/ethnicity, insurance, baseline year, hypertension, diabetes, current smoking, statin use, and systemic steroid use. CHD: coronary heart disease, HR: hazard ratio, CID: chronic inflammatory disease, HIV: human immunodeficiency virus, RA: rheumatoid arthritis, SLE: systemic lupus erythematosus, SSc: systemic sclerosis, IBD: inflammatory bowel disease.
